# Charge Distribution and Lithium Oxide Stability Modeled by Reactive Force Field

**DOI:** 10.1021/acs.jpca.5c03998

**Published:** 2025-10-13

**Authors:** Vjeran Gomzi, Jakov Juvančić

**Affiliations:** 112586 University of Zagreb, Faculty of Electrical Engineering and Computing, Unska 3, Zagreb 10 000, Croatia

## Abstract

Understanding the reactive properties of lithium and its oxides plays an important role in the modeling and design of lithium batteries. For the investigation of reasonably large structures, the use of molecular dynamics is usually the method of choice because of its calculation efficiency. The shortcoming of this approach is that the electron distribution is approximated by parameters obtained semiempirically or approximated at different levels from first-principles calculations. A novel method based on Kohn–Sham density functional theory, approximated to the second order (ACKS2), for modeling the charge distribution has recently been introduced. The method resolves two major problems from which the previous electronegativity equilibration method suffers, although some shortcomings remain. Here, we first verify the effect that the charge calculation method has on theoretical reproduction of the atomic charges obtained by the model, and then proceed to optimize the force field parameters in an attempt to alleviate the problems perceived. The newly trained ACKS2 reactive force field is validated and shown to be able to reproduce the structure and charge distribution of the lithium crystal and lithium-oxide crystal slabs enclosed by the vacuum layer.

## Graphical abstract

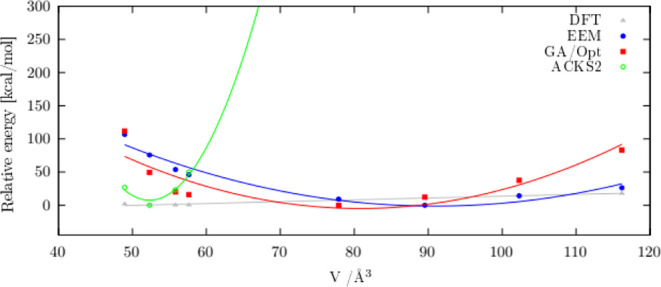

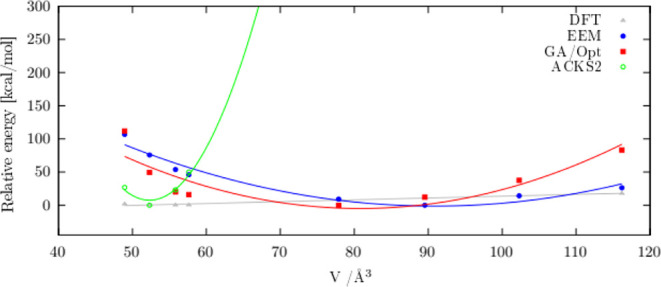

## 1 Introduction

Understanding of lithium and its oxidation processes is of interest in the design and development of lithium-based batteries, especially those based on lithium–air technology.^1^ Despite their advantages (energy density similar to gasoline) over conventional Li-ion batteries, their efficiency is still lower, so the catalytic approach is commonly used for possible improvements. Investigation of a large reaction space is often much more efficient with the help of *in silico* methods. Of a number of methods available, molecular dynamics (MD) using the reactive force field approach, as applied in the *ReaxFF* package, is a promising method optimizing simulation capabilities and calculation efficiency.^2,3^ The reactive force fields have an advantage over more traditional MD approaches in that it is possible to use the same set of atom parameters for different bonds, thus being able to model, to some extent, chemical reactions. Relatively recent activity in the application of *ReaxFF* MD to lithium-based batteries has been reviewed in ref 4, showing considerable interest and establishing a firm foundation for efforts toward a reliable reactive force field for lithium-oxide MD simulations.

The reactive force field (FF) based on the electronegativity equalization method (EEM) charge model has been optimized and used with reasonable success.^5^ This method is included in the original implementation of the *ReaxFF* code.^6^ Further reparametrization protocols for EEM-based force fields are being actively developed.^7^ The EEM method, however, suffers from two important theoretical and practical limitations: (1) the method predicts partial charges even at dissociation of the atoms, and (2) the dipole polarizability scaling with system size is predicted to be cubic, while dielectric systems exhibit a linear scaling in the macroscopic limit. Several attempts at alleviating these issues were devised, sometimes lacking a firm theoretical basis. A comprehensive list may be found in ref 8. The charge distribution, and especially electron transfer reactions, are thus expected to be described more accurately within *ReaxFF* MD simulations using the novel atom-condensed Kohn–Sham DFT to the second order charge interaction method (ACKS2), which is constructed to address these deficiencies of the EEM.^8^ However, the use of the novel method in the *ReaxFF* implementation requires reoptimization of the whole force field.

The reactive force field based on the ACKS2 charge distribution model, targeted at modeling cracks in the Li_2_O structures, has been developed and tested by O’Hearn et al. in 2020.^2^ The method is optimized for modeling Li_2_O and Li_2_O_2_ crystal structures. It has, however, been reported that in lithium–air technology, the Li_2_O_2_ film formation reducing the efficiency of the cell is related to the solvated LiO_2_.^1,9^ The lithium-superoxide species is of special interest in the potential closed battery design, in which no additional gases are permitted to contribute to the reaction. Such a model system has been recently investigated.^10^ Also, there is evidence that all three oxide species—LiO_2_, Li_2_O_2_ and Li_2_O—are present and important in solid electrolyte lithium batteries during film growth on Mo_3_P cathode, as the reaction
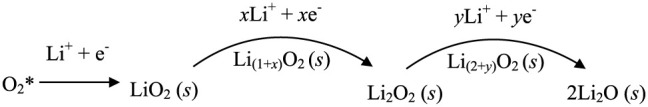


is found to take place.^11^ Furthermore, nonstoichiometric Li_*x*_O_*y*_ compounds are expected to exist at crystal or nanoparticle boundaries (*ibid.*). Thus, the interest is to verify the available force field(s), and subsequently, the aim is to develop a force field able to model all the stable lithium-oxide structures.

## 2 Methods

The work is composed of two related parts. In the first part, we investigate the effect of the use of the charge interaction model (EEM or ACKS2) on the prediction of charge distribution in lithium oxides and compare these with theoretical results obtained from density-functional theory (DFT). Based on insights from this comparison, in the second part of the work, we develop the ACKS2-based force field capable of reasonably reproducing experimentally observed crystals in all lithium oxides (i.e., LiO, Li_2_O, LiO_2_, Li_2_O_2_, and LiO_3_). The work included choice of parameters and the choice of the training set for optimization, the optimization procedure itself, and finally the validation of the developed force field.

### 2.1 Atomic Charge Distributions of EEM- and ACKS2-Based Force Fields

The foundation of the EEM method is set by Sanderson in 1976, stating that charge on atoms in a molecule may be obtained from the difference between the electronegativities of the free atoms and atoms in molecules.^12^ In molecules, the atoms acquire mean electronegativity as the geometric mean of the atoms in the molecule, i.e.

1 $$\chi_{gm}{=}^{n}\sqrt{\overset{n}{\underset{i=1}{\prod }}\chi_{i}}$$

The Sanderson’s postulate is subsequently theoretically established, relating it to DFT,^13,14^ and used by Mortier et al.^15^ to develop a practical implementation for the calculation of partial atomic charges. The point-type charge interactions were replaced by interactions of 1*s* Slater-type orbitals in the works of Rappe and Goddard^16^ and Njo et al.^17^ Such a method variant is implemented in the *ReaxFF* code.^8,18^

The most general energy decomposition used in the full *ReaxFF* implementation may be written as

2 $$E_{\mathrm{system}}=E_{\mathrm{bond}}+E_{\mathrm{overcoordination}}+E_{\mathrm{angle}}+E_{\mathrm{torsion}}+E_{\mathrm{vdWaals}}+E_{\mathrm{charge}}$$

The last term is the long-distance electrostatic interaction, depending on the charge distribution model. The physical interaction behind this term is the Coulomb potential, to which additional terms, such as exchange-correlation energy, are added within the DFT framework. For the conventional EEM, however, these are discarded, so the following formula for electron interaction energy is used^17^

3 $$E_{\mathrm{el}}=\frac{1}{4\pi \epsilon_{0}}\underset{ij}{\sum }\frac{q_{i}q_{j}}{r_{ij}}$$

4 $$E_{\mathrm{EEM}}=\min_{q_{i}:\sum q_{i}=q_{\mathrm{tot}}}\left[\overset{N}{\underset{i=1}{\sum }}\chi_{i}q_{i}+\frac{1}{2}\underset{i=1,j=1}{\sum }\eta_{ij}q_{i}q_{j}\right]$$

where χ_*i*_ and η_*ij*_ are intrinsic atomic electronegativity and atomic hardness, which are obtained by solving the linear set of equations^8^

5 $$-\left[\begin{array}{ll} \vdots & \vdots \\ \eta & -d\\ \vdots & \vdots \\ -d^{T} & 0 \end{array}\right]\left[\begin{array}{l} \vdots \\ \Delta \\ \vdots \\ \mu_{\mathrm{mol}} \end{array}\right]=\left[\begin{array}{l} \vdots \\ \mu \\ \vdots \\ \Delta_{\mathrm{tot}} \end{array}\right]$$

In eq 2, partial charges (Δ) and total chemical potential (μ_mol_) are calculated knowing the atomic hardness matrix given as *d*, and *d* ^T^ are column and row matrix filled with ones, while μ is the column of atomic electronegativities. The hardness matrix is defined by calculated Taper correction of the interatomic distance and γ_*i*,_ which are read from the force field for the atomic species. The above linear system, i.e. the leftmost matrix, is of the size (*N*+1) × (*N*+1), where *N* is the number of atoms.

6 $$\begin{array}{l} \frac{\eta_{ii}}{2}=\eta_{i}\\ \eta_{i}j=\mathrm{Tap}\left(r_{i}j\right)\frac{1}{4\pi \in_{0}}\frac{\left(q_{i}q_{j}\right)}{{[r_{i}j^{3}+{\left(\gamma_{i}\gamma_{j}\right)}^{-3/2}]}^{1/3}} \end{array}$$

In contrast, in the ACKS2 implementation, energy is calculated as (*ibid.*)­

7 $$E_{ACKS2}=\underset{q_{i}:\sum q_{i}=q_{tot}}{\min}\left[\sum_{i=1}^{N}\chi_{i}q_{i}+\frac{1}{2}\sum_{i=1,j=1}\eta_{ij}q_{i}q_{j}+\underset{u_{i}:\sum u_{i}=0}{max}\left[\sum_{i=0}^{N}u_{i}\left(q_{i}-q_{i}^{0}\right)+\frac{1}{2}\sum_{i=1,j=1}^{N}X_{ij}u_{i}u_{j}\right]\right]$$

Where the first two parts are the same as in the EEM approach, and the last two are ACKS2 corrections, in which figure two new quantities: the Kohn–Sham (KS) expansion coefficients, *u*_*i*_, and the softness parameters *X*_*ij*_ directly related to the dielectric response. The latter are calculated by the expressions

8 $$X_{ij}=X_{\mathrm{soft}}{\left(\frac{2r_{ij}}{C_{i}+C_{j}}\right)}^{3}{\left(1-\frac{2r_{ij}}{C_{i}+C_{j}}\right)}^{6}\mathrm{if}r_{ij}<\frac{C_{i}+C_{j}}{2},\mathrm{otherwise 0}$$

9 $$X_{ii}=-\overset{N}{\underset{j=1,j\neq i}{\sum }}X_{ij}$$

where *X*_soft_ and *C*_*i*_ are softness parameters read from the force field. The change in KS effective potentials *u*_*i*_ are obtained by solving the ACKS2 linear system in the same calculation that is used for obtaining the partial charges, electronegativity of the molecule, and the Lagrange multiplier, λ_U_

10 $$-\left[\begin{array}{llll} \eta_{e} & -d & -I_{M} & 0\\ -d^{T} & 0 & 0 & 0\\ -I_{M} & 0 & X_{S} & -d\\ 0 & 0 & -d^{T} & 0 \end{array}\right]\left[\begin{array}{l} \Delta \\ \mu_{\mathrm{mol}}\\ U\\ \lambda_{U} \end{array}\right]=\left[\begin{array}{l} \mu \\ 0\\ 0\\ 0 \end{array}\right]$$

where the problem is obviously an extension of the EEM, and the matrix is now of the size (2*N* + 2) × (2*N* + 2). The additional set is, however, relatively sparse, so the calculation burden is usually not comparatively larger, as will be shown below.

The *Python 3* routine for calculation of interaction energy and charge distribution using the reactive force field, either by application of the EEM or ACKS2 charge interaction model, was modified and partially rewritten in the course of one of the author’s bachelor’s work (JJ) based on available code and information.^8,18,19^ The aim of this part was to understand the practical implementation of the underlying theoretical approach. The program uses a simplified energy calculation, neglecting bond-order and other interatomic interactions in eq 2 and is used mainly for calculation of charge distribution based on the given structure. The periodic calculation capability is not implemented.

The charge distribution for test structures is also calculated by applying density-functional theory calculations using the Natural Population Analysis (NPA) charge method^20–22^ in *Gaussian* 16 (G16)^23^ with B*3LYP/6–31+G* density functional/basis set^24^ and *Abinit* using ultrasoft periodic potential,^25^ as well as the Quantum Espresso (QE)^26^ and the complete *ReaxFF* (*ereac*) packages^27^ run on the Supek HPC cluster at SRCE, University of Zagreb Computation Center. The comparison of the results obtained by the developed *Python* code and other approaches is then used for the investigation of the dependence of the charge reproduction on several parameters, such as the method/force field, but also on the periodic or nonperiodic code (*G16*, *QE*, *Abinit*, and *ReaxFF*) on the optimization or crystal constraints on the structure, and finally on the number of terms included in eq 2 in modeling the charge distribution of the structure. Full data on structures and charges used in this part are given in the Supporting Information 1, parts A and B. The Li/O *ReaxFF* EEM and ACKS2 FFs used for calculations were taken from refs 5 and 2 respectively.

The e-reactive approach (using ACKS2 FFs) is still in the experimental phase, so we wanted to gain knowledge on its performance and put it in relation to the more conventional approach (EEM) that is still being actively used and trusted to provide reliable results.^28^

### 2.2 Reoptimization and Validation of the ACKS2 Force Field for Li–O Crystals

In the second part of the work, we reoptimized the ACKS2 force field developed by O’Hearn et al.,^2^ as it was noticed that the previously optimized FF had problems reproducing the LiO_2_ slab (crystal structure with vacuum layer). The additional parameters were included in the optimization attempt, as this was expected to benefit the results. A full list of optimization parameters is shown as highlighted differences in Figure S3. All the parameters were first optimized (and reoptimized) using the parabolic parameter optimization method implemented in the original *ReaxFF* standalone code.^6,27^ As a result of this process, the obtained FF did not show the expected improvements (data not shown), so a decision to attempt to obtain the optimized force field is made. Thus, the following approach is designed: the force field is optimized by the wrapper *Python* routine governing the general optimization process, involving the *Optuna* ^29^ study using the Nondominated Sorting Genetic Algorithm,^30^ and for updating the *ReaxFF* force file (*ffield*) with the new trial parameters. This routine also calls a Linux bash shell script for running external software for specific tasks: *ereac* for *ReaxFF* calculation, *calculate_rmsd* ^31^ for calculation of spatial structure differences between the training set and *ReaxFF* trial and comparison of training charges and energies with the charges and energies obtained by *ReaxFF* as calculated in the *fort.56* file (charges) and *xmolout* (energies). The code strongly relies on a specific setup on the cluster used for calculations and is being developed to fit a more general application. A detailed description of the setup and the pseudocode is given in Figure S2, and the full scripts are available from the corresponding author upon request. The training set consisted of crystal structures of all the lithium oxides (i.e., Li_2_O, LiO_2_, Li_2_O_2_, and LiO_3_), their supercell structures, as well as the crystal structures with vacuum layers added in each of the cell directions (*a*, *b*, or *c*, one at a time). The crystal structures are obtained from the Crystallographic Open Database archives,^32,33^ while supercells and other derived structures used as training set are also available in the Supporting Information 1, part C of this article.

Upon the optimized FF being obtained, it is applied to MD calculation of 1000 steps of 0.25 fs intervals of the four lithium-oxide crystal species (their 2 × 2 × 2 unit cells) at 1 K for validation of charge and geometry reproduction. Furthermore, the MD simulation of the pristine LiO_2_ crystal (7 × 7 × 7 repeated unit cells) has been performed with all of the cell dimensions symmetrically increasing/decreasing at a rate of 0.04 Å/0.25 fs using the *ReaxFF* code. The energies of the same structures have been calculated using the periodic DFT approach, applying the *PBE* exchange-correlation pseudopotentials on O and Li available at refs 34,35 . Prior to calculation of energy, the periodic structure is relaxed within a fixed cell size (full QE inputs are provided for reference in Supporting Information 1, part D). In this way, the equation-of-state (EOS)-like diagrams of volume dependence on energy were used for FF validation.

## 3 Results

In the first part of the work, the modified reproduced *Python* code is applied on all the lithium oxide unit cells (Li_2_O, LiO_2_, Li_2_O_2_, and LiO_3_) at their crystal positions and as isolated supercell structures using available force fields.^2,5^ As most of the energy contributions were missing in the test code, the comparison of the applied FF’s and EEM/ACKS2 methods was also targeted at restricted geometries in their crystal positions, as well as to the energy-minimized *i.e.* molecular-mechanics (MM) optimized structures obtained by the use of all the parameters in the energy expansion (eq 2) through the use of the full *ReaxFF* code.

The full list of calculations performed using the single-unit cell structures is as follows.

### Single Unit-Cell Considerations

(1) *Python* code calculation of the unit cell at their crystal structure using ACKS2 FF (pyA)

(2) *Python* code calculation of the unit cell at their crystal structure using EEM FF (pyE)

(3) Full *ReaxFF* code calculation of the unit cell at their crystal structure using ACKS2 FF (RrA)

(4) Full *ReaxFF* code calculation of the unit cell at their crystal structure using EEM FF (RrE)

(5) Full *ReaxFF* code calculation of the unit cell at the optimized crystal structure using the ACKS2 FF (RoA)

(6) Full *ReaxFF* code calculation of the unit cell at the optimized crystal structure using the EEM FF (RoE)

All charges predicted by the the reactive force-field method, using both the EEM and ACKS2 approaches, are compared with respective theoretical calculations at the *B3PW91/Lanl2DZ* DFT level using the *G16* program package run on the SRCE HPC cluster. Squares of differences of predicted charges at respective atoms in all the investigated structures are summed, and the root-mean square (RMS) error is calculated as

11 $$\sigma =\sqrt{\frac{1}{N}\overset{N}{\underset{i=1}{\sum }}{\left(q_{MD,i}-q_{DFT,i}\right)}^{2}}$$

where *q*_MD_ and *q*_DFT_ are charges obtained by MD and DFT approaches, respectively.

The comparison of RMS error of charge distribution (in relative units of unit charge, *e*) for the single unit cell calculations is presented in the charts below, showing the dependence of charge RMS error on the calculation protocol applied, as shown in Figure 1. It should be noted that here all the data are compared to *G16* NPA charges based on the single unit cell of the respective lithium oxide fixed in their crystal positions. Although it is obviously not expected to perfectly model the distribution in the actual periodic system, several interesting and important conclusions may be drawn based on this. Also, as explained in the introduction (and references therein), processes on electrodes are expected to occur locally, so the model of local formation of crystallites of specific oxide species may also be of interest.

**Figure 1:**
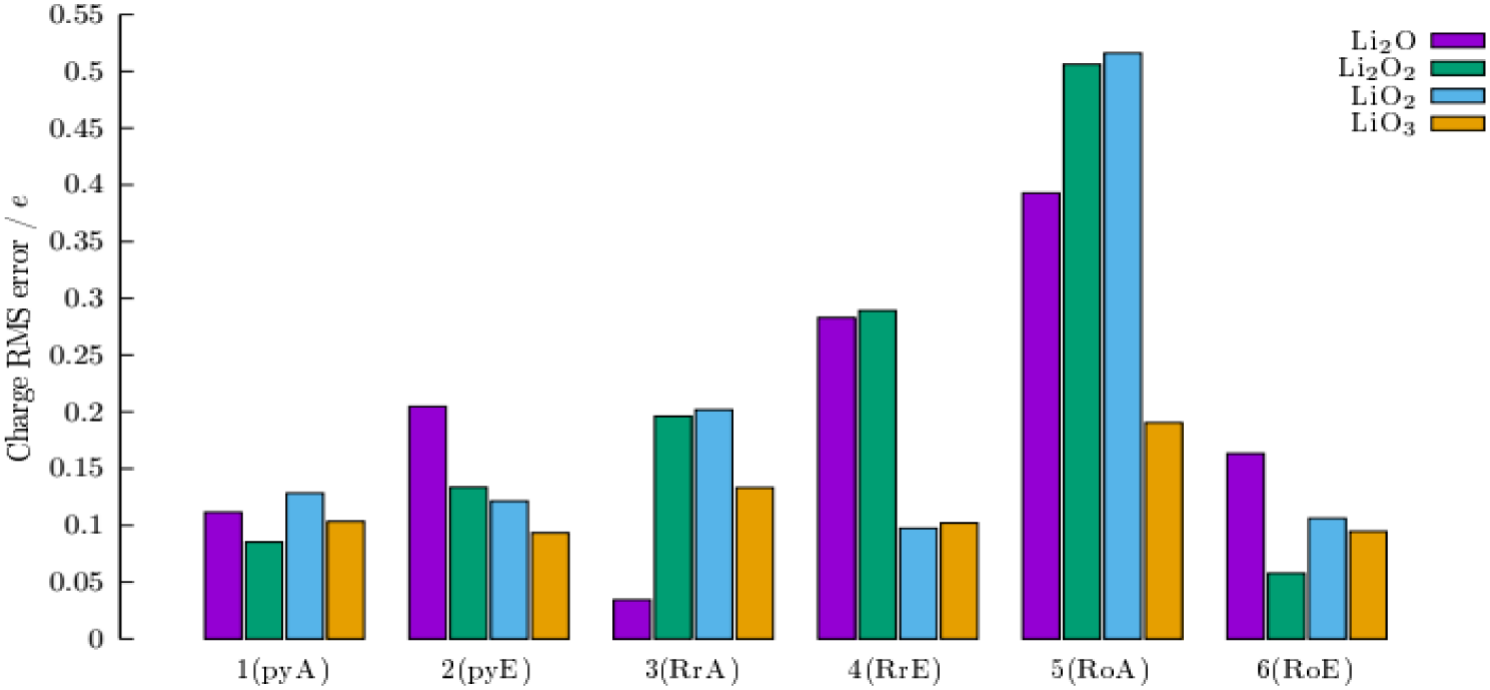
Comparison of differences in charge distributions from DFT theoretical values for different calculation protocols for all lithium oxide single-unit cell structures.

In Figure 1, we may observe several interesting features:

1. simple calculations of structures as small as the unit cell (6–9 atoms), fixed on their crystal positions, reproduce reasonably well the corresponding DFT charges. No other interactions are involved apart from electronic contribution, modeled by EEM or ACKS2 (subcharts 1 and 2 above).
2. Full *ReaxFF* code on such unit cell structures performs reasonably well (subcharts 3 and 4). Discrepancy of charges from DFT data is different for the EEM and ACKS2 for specific oxides.
3. Optimization (thus also including the crystal environment in the *ReaxFF*) leads to a large deviation in predicted charges for the ACKS2 model compared to the EEM model, which reproduces very well the individual unit cell calculation (subcharts 5 and 6).

From the last observation, it may be inferred that the long-range interaction of the ACKS2 calculation, as well as the charge distribution on neighboring atoms, significantly modifies the local charges in the central unit cell. This is to be expected; therefore, this behavior of the ACKS2 model indirectly supports its expected reliability in charge distribution reproduction. Discrepancy in these calculations, however, was the motivation for additional FF optimizations below.

Full details of additional performed calculations involving a 2 × 2 × 2 supercell and description of results from this cross-comparison are given in the Supporting Information 2 (denoted as section B). The most important conclusion from this part of the work is the observation that additional geometry optimization (MM energy minimization) seems to introduce a discrepancy in reproduction of the charges. This may stem either from suboptimal parameters obtained in the course of the previous work or the lack of inclusion of some parameters in the optimization process. (Alternatively, these additional parameters may also be related to the parameters included in the optimization, which would in turn require their reoptimization.) In whichever case, based on this insight, a decision is made to subsequently attempt optimization of a larger parameter set to develop the ACKS2-based reactive FF.

To complete the comparison of EEM and ACKS2 methods using the model reduced *Python* code, we note the relative time difference required for calculation of charges (and energies) of the 768-atom LiO_2_ supercell crystal structure. The times required for calculations (on single Pentium Core i5, eighth. gen) using the two charge methods are

EEM⁡3.461⁡s

ACKS2:⁣7.894⁡s

Thus, although the size of matrices used in calculations (see Methods section, eqs 15 and 12) would suggest, because of *O*(*n* ^3^) dependence of the number of operations on matrix size, that the calculation for ACKS2 would be 2^3^ = 8 times longer for the same system, the calculation time for the ACKS2 approach is approximately only about twice as long for the structure used. It should be noted that this is only used for a demonstration and by no means presents the investigation on the resource requirements.

In the following work, we first tested the stability of the large 864-atom Li crystal slab, to which a 25 Å vacuum layer was added, under MD simulation using the two available reactive force fields. The stability may conveniently be validated by the number of fragments into which the initial structure decomposes (or not) during *ReaxFF* MD simulation, Table 1 (It should be noted that the temperature of the thermostat used in the simulation is held at 1 K, so to minimize thermal kinetic effects. The simulation is performed for 1000 steps of 0.25 fs).

**Table 1:** Comparison of the Number of Fragments as a Measure of Crystal Shape Stability Reproduced Appliyng the Respective FF to the 864 Atom Li Crystal Slab with a 25 Å Vacuum Layer after 1000 0.25 Fs MD Iterations^a^

ACKS2		EEM		GA/Opt	
*(Nfrags) × formula*	*molecular mass*	*(Nfrags) × formula*	*molecular mass*	*(Nfrags) × formula*	*molecular mass*
4 × Li72	499.7520	1 × Li864	5997.0240	1 × Li864	5997.0240
7 × Li12	83.2920				
1 × Li360	2498.7600				
33 × Li2	13.8820				
1 × Li60	416.4600				
1 × Li6	41.6460				
Total number of molecules: 47		Total number of molecules: 1		Total number of molecules: 1	
Total number of atoms: 864		Total number of atoms: 864		Total number of atoms: 864	

a The result obtained by the developed force field GA/Opt is also included in the table for reference.

The ACKS2 Li/O FF implementation is thus not completely satisfactory, as we are interested in the force field able to model reactions and oxidation changes on the Li electrode. This was the main motivation for the subsequent work in the following part of our work.

Based on considerations concerning theoretically reproduced charges in the former part of the work, it was decided to increase the number of parameters considered in ref 2. Previous optimization considered 28 parameters, while we augmented this set to 40. The additional parameters are included since the relatively inaccurate charges obtained if geometry optimization is applied in *ReaxFF* suggestthat some of the geometry parameters may be the reason for this discrepancy. The parabolic optimization process implemented in the standalone *ReaxFF* code is both laborious and lengthy, although it is a relatively safe method for optimization of a considerable number of optimization parameters (as much as 40 here). However, it fails if the parameters are related. Initially, the parameters were optimized using this method successfully only upon reversing the order of parameters to optimize (from those describing less important energy contributions to those that are the most important, general parameters traditionally stated at the beginning of the FF definition, representing probably the level of parameter importance and their effects on other subsequent parameters: we had to optimize from the least important to the most important ones). The optimization finished after about 30 days (running as a single process), but the optimized force field did not considerably affect stability or charge accuracy reproduction for vacuum slabs (data not shown). So, the alternative *Genetic Algorithm* optimization using *Optuna* (GA/Opt) method (as described above) was applied. As this method obtains optimized FF files in less than 3 days (running as a single process), we added 8 more parameters to the set. A total of 48 parameters were optimized using *Optuna* and a training set of crystal structures of the four oxides, free Li crystal, the O_2_ molecule, as well as crystal slabs with a vacuum layer of about 20 Å added at each of the crystal axes. Training set also included NPA *G16* DFT charges for 2 × 2 × 2 unit-cell structures of all the crystals. The complete training set is given in the Supporting Information of this article. The relative weights of the training sets (structures, charges, or energy differences) were initially set to be consistent with measurement units. However, it is found that the charge is reproduced well only if the structure is also reproduced with reasonable fidelity. Also, the relative weights of energies for the Li crystals of different volumes and the four lithium-oxide crystal structures were also important. So the relative weights for

w[structurediscrepancy(Å)]:w[chargedifferencee(e)]:w[energy(kcal/mol)]

were after a few initial tries, set to

1:10:0.0001

The population size is taken to be 3000 (as suggested to scale with the square of the number of parameters), the mutation probability of 0.5 and 1500 optimization iterations. The resulting FF is validated using an EOS-like approach for the LiO_2_ crystal by comparison to the respective data for DFT, ACKS2, and EEM values, as well as RMSE of structures and charges for 2 × 2 × 2 supercells of the four oxides.

The application of the considered FFs to 2 × 2 × 2 supercells of the considered lithium-oxide structures is compared in Table 2. The structures are MD simulated for 1000 steps of 0.25 fs at 1 K. Charges are compared to *G16* DFT NPA values on the same structures at their crystal positions.

**Table 2:** Comparison of the Charge RMSE and Position RMSE for the ACKS2, EEM, and Newly Developed GA/Opt FF

RMSE	ACKS2	EEM	GA/Opt
Charge/e			
Li_2_O	0.35	0.53	0.35
Li_2_O_2_	0.47	0.81	0.36
LiO_2_	0.95	0.32	0.25
LiO_3_	0.89	0.32	0.24
Structure/Å			
Li_2_O	1.98	1.18	0.24
Li_2_O_2_	1.57	1.53	0.75
LiO_2_	1.45	1.74	0.09
LiO_3_	1.35	1.44	0.10

Validation is further conducted as described in the Methods section, considering the energy change dependence on the cell volume of the LiO_2_ crystal. EOS-like diagrams are obtained by selecting three structures corresponding to reduced volumes and four structures corresponding to increased cell volumes and comparing MD energy profiles to the QE periodic DFT calculations for the same cell dimensions (Figure 2).

**Figure 2:**
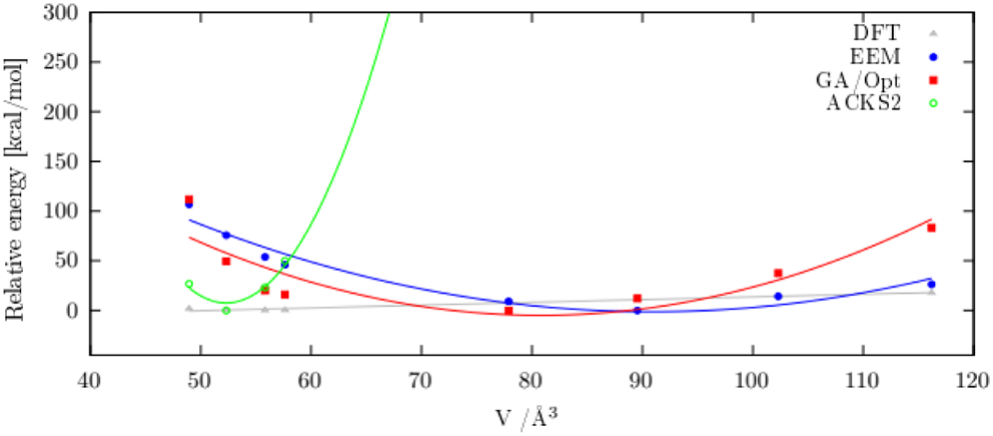
Energy changes upon cell volume modification for the LiO_2_ crystal structure.

Superior performance of the newly developed GA/Opt FF is shown in Table 2 and Figure 2. The behavior is expected to be generally reasonable and not merely the result of overoptimized a specific structure, as especially seen from Figure 2, where both the EEM and GA/Opt FFs show relatively mild volume dependence, in agreement with DFT calculations, which are supposedly critical for the accurate charge and stability description of Li/O structures. Furthermore, it is seen that we obtained superior crystal structure reproduction using the training set dominantly relying on the quality of the charge reproduction (Table 2).

**Figure 3:**
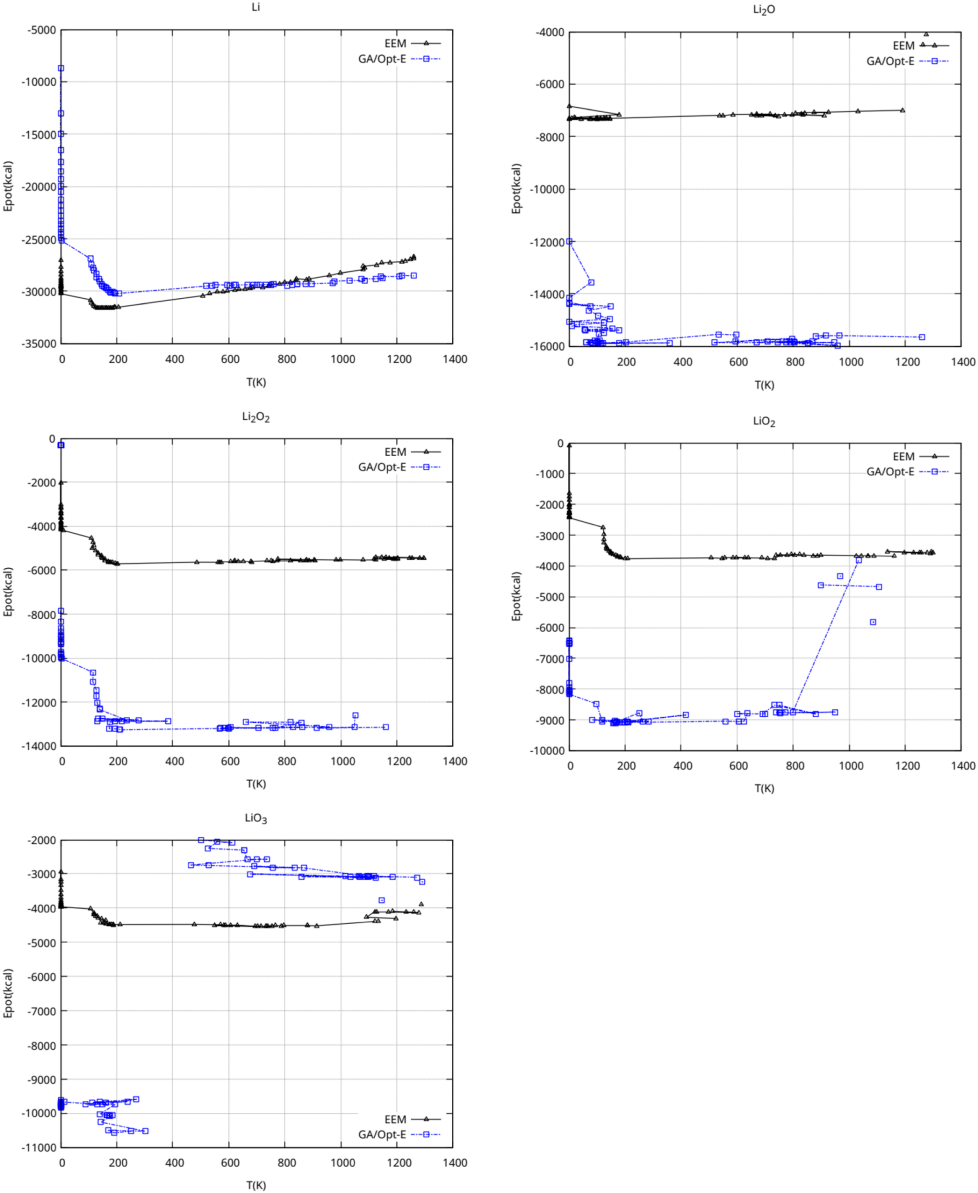
Comparison of GA/Opt-E and EEM results. Energy changes upon simulation temperature modification for all considered crystal structures.

### Final Considerations: Force-Field Stability

To test possible use of the developed GA/Opt FF, we applied it to the calculation of a Li crystal slab with a vacuum layer of 24 Å, in which 10 O_2_ molecules are added (Figure 5, structure available in the Supporting Information 4).

**Figure 4:**
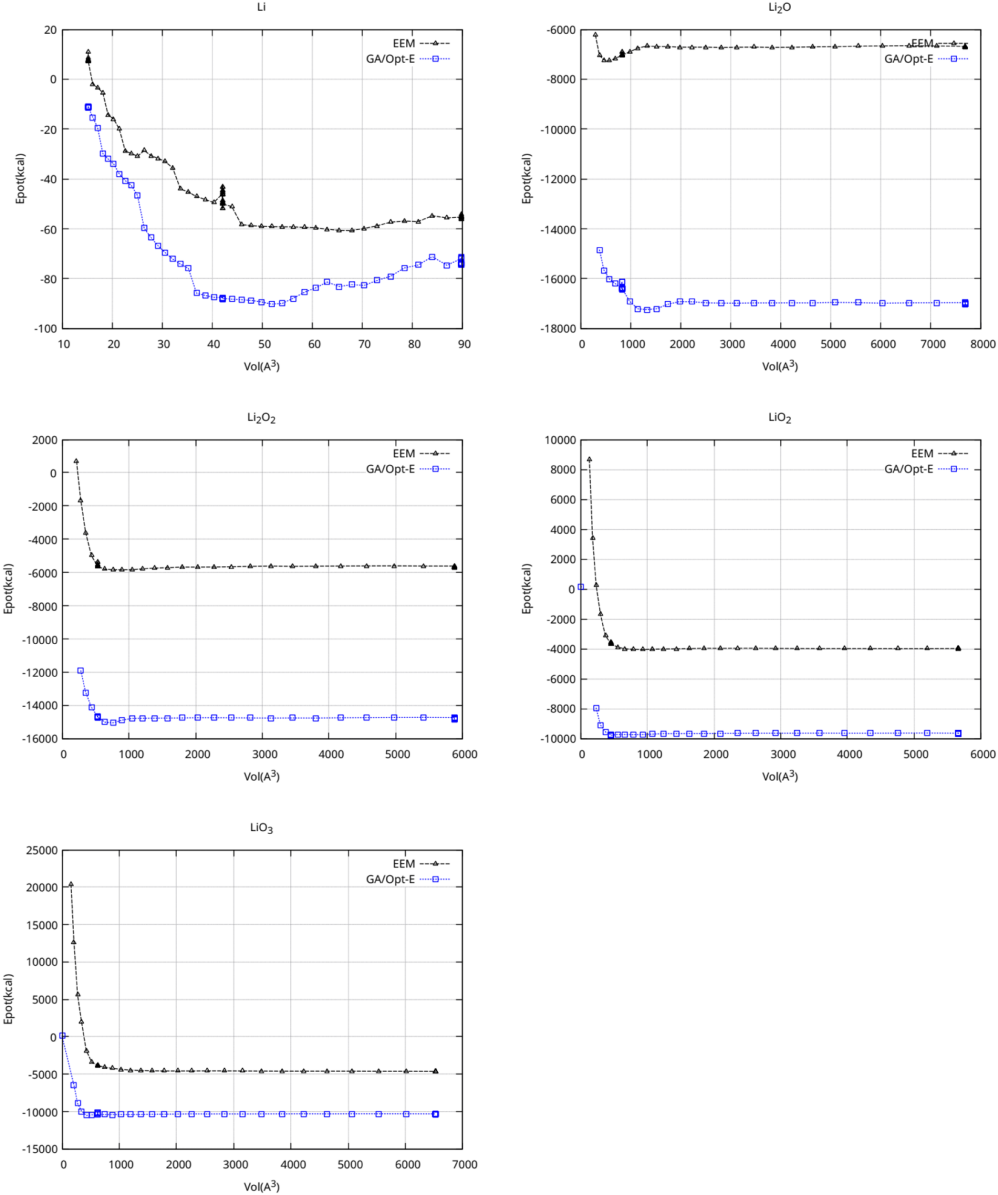
Comparison of GA/Opt-E and EEM results. Energy changes upon unit cell volume modification for all considered crystal structures.

The structure is simulated for 50,000 steps of 0.25 fs each using the NPT barostat, with the temperature rising from 1 to 10, 50, and 100 K and then back, in 10,000 step intervals. The reactivity of pure Li with oxygen at room temperature is known to be small,^36^ so the effect, should there be any, should be only a small fluctuation from the initial structure. A comparison of the two simulation studies, using EEM and GA/Opt, shows that the latter does not fulfill these assumptions, as the structure decomposes at the initial step. The structure modeled by EEM FF seems to be almost completely inert throughout the simulation, and no large structural modifications are observed. The ACKS2 FF already fails to simulate pure Li crystal at 1 K and was thus not included in this comparison.

In an attempt to improve Li crystal stability upon rising temperature and long simulation times, we included additional components in the training set. Namely, these were the pure lithium crystal DFT energies calculated using the same periodic approach implemented in QE and applied the same PBE basis set as already explained in the Methods section for several lithium crystal unit cell volumes. The same is done for the Li_2_O 72-atom supercell structure. In this way, we formed an analogue of Li and Li_2_O crystal EOS to which we trained our basis set. The initial force field for this additional training is taken to be GA/Opt previously optimized FF. As new structures are added to the training set, relative weights of the structures and of the energies were modified, as well as the relative weights of different training constraints, as following:

w[structurediscrepancy(Å)]:w[chargedifference(e)]:w[energy(kcal/mol)]

was set to

1:10:1

where the energies and structures of oxides from the previous and new training sets are further scaled as given in the *enewt.wt* and *gemwt.wt* sections of Supporting Information 2. This was a part that had to be chosen with utmost care, and the current optimized parameters are obtained after a few initial test trials.

The final training and validation sets can be summarized as follows (where simulation of the lithium-crystal slab is used as the final stability test and is not added to this summary):

Optimized ReaxFF Parameters: 48.

Training (Optuna Optimization, ReaxFF MD Energy Minimization): GA/Opt: 27 crystal and supercell structures (total of 1060 atoms), 62 charges, and 3 energy relations.

GA/Opt-E: 52 crystal and supercell structures (total of 2392 atoms), 62 charges, and 26 energy relations.

Initial Checks (Geometry and Charge Reproduction RMSE: Supercell structures: (Li_2_O)_24_ (Li_2_O_2_)_16_ (LiO_2_)_16_ (LiO_3_)_16_.

Respective charges on atoms obtained from DFT Natural Population Analysis: 72, 64, 48, and 64 entries.

Validation and Stability: Comparison of volume-energy and temperature-energy profiles using the new GA/Opt-E ACKS2 FF with the EEM FF for both volume increase and decrease (all unit cell dimensions modified at the rate 0.04 Å/0.25 fs in a maximum of 20,000 steps) and for simulation at rising temperature (from 1 to 1300 K and back to 100 K in a maximum of 50,000 steps of 0.25 fs each).

Comparison of reproduction of structures and charges for the novel GA/Opt-E and EEM FF upon modification of temperature damping constant (1 fs, instead of otherwise systematically used 0.25 fs).

Such an approach yields a new force field which shows stability upon prolonged MD simulation time and upon temperature rise. The atom positions during simulation even show some crystal size fluctuations, which is expected upon warming, somewhat in contrast to the EEM results. (The trajectory is available in the Supporting Information 4). The resulting force field, denoted as GA/Opt-E, mostly retains all the desirable features of the GA/Opt FF obtained previously, as seen in Table 3.

**Table 3:** Comparison of the Charge RMSE and Position RMSE for ACKS2, EEM, and the Developed GA/Opt and GA/Opt-E FFs

RMSE	ACKS2	EEM	GA/Opt	GA/Opt-E
Charge/e				
Li_2_O	0.35	0.53	0.35	0.39
Li_2_O_2_	0.47	0.81	0.36	0.43
LiO_2_	0.95	0.32	0.25	0.23
LiO_3_	0.89	0.32	0.24	0.28
Structure/Å				
Li_2_O	1.98	1.18	0.24	0.45
Li_2_O_2_	1.57	1.53	0.75	0.24
LiO_2_	1.45	1.74	0.09	0.28
LiO_3_	1.35	1.44	0.10	0.26

In Figures 3 and 4, the predicted *E*(*T*) and *E*(*V*) dependencies for the newly developed GA/Opt-E FF are compared to the EEM results. This comparison shows that the energy and temperature dependencies of the FF optimized in this work are compatible with the already established Li/O EEM FF. This, in turn, establishes important arguments in favor of its reasonable behavior.

We see from Table 4 that the developed field still shows superior performance under modified simulation parameters (here, a time step of 1 fs was used instead of 0.25 fs used otherwise). Other comparisons with EEM results (*E*(*V*) and *E*(*T*) dependencies analogous to those in Figures 3 and 4) for this time step modification show the same stability of predictions (data not shown).

**Table 4:** FF Stability Test: Comparison of the Charge RMSE and Position RMSE for the EEM and Newly Developed GA/Opt-E FFs for a Time Step of 1 Fs

RMSE	EEM	GA/Opt-E
Charge/e		
Li_2_O	0.52	0.45
Li_2_O_2_	0.27	0.24
LiO_2_	0.28	0.28
LiO_3_	0.25	0.26
Structure/Å		
Li_2_O	0.68	0.58
Li_2_O_2_	0.85	0.60
LiO_2_	0.40	0.30
LiO_3_	0.37	0.47

As stated in the introduction, the motivation for this research is development of the *ReaxFF* method to simulate processes in a lithium–air battery. The actual temperatures in batteries are around or higher than the room temperature (300 K), thus the application of the EEM and GA/Opt-E FFs in this regime has also been tested using the NPT barostat with the same parameters as above, running the MD simulation for 50,000 steps of 0.25 fs each at 300 K. (It should be noted that the lithium crystal melting temperature is 453 K). From the data presented in Figure 5, it is shown that the GA/Opt-E FF predicts lithium structural changes, although this is mostly visible on the slab surfaces, while lithium modeled with the EEM FF is disturbed much more evenly. This may relate to the long-distance interactions modeled differently in the two (ACKS2 or EEM) approaches. In view of these differences in behavior, neither of the evaluated force fields should be considered completely safe for application in the simulation of oxidation processes at these temperatures until further thorough verification by comparison with experimental data is provided.

**Figure 5:**
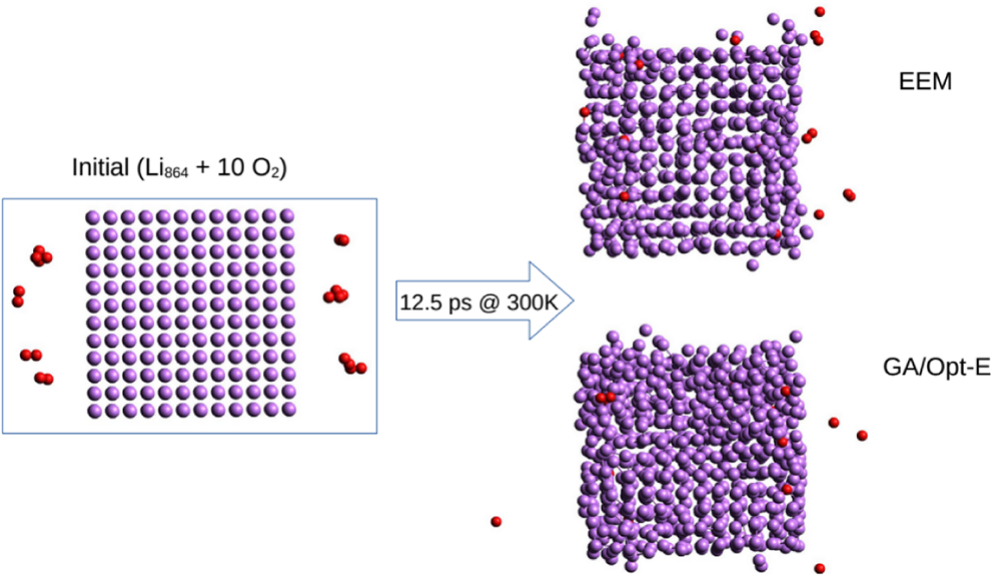
Simulation of the lithium-crystal slab using the NPT thermostat at 300 K for 12.5 ps. Comparison of the EEM and newly developed GA/Opt-E FFs.

Some remarks based on considerations of the available theoretical and experimental data on lithium and its oxides, and the simulation results presented (*E*(*V*), *E*(*T*) patterns, and 10 O_2_ + Li_864_ system), are in order, nevertheless:

1. The EEM FF is well-established standard for *ReaxFF* calculations.^5^ The comparison of the energy patterns obtained using GA/Opt-E upon modifying volume and temperature with EEM results is used for validation. It should be noted that the force field is trained only at one temperature (293 K), and using explicitly only Li and Li_2_O energy-volume dependence. Thus, the reasonable *E*(*V*) dependence of the LiO_2_ and Li_2_O_2_ and the *E*(*T*) dependence for all the structures are supporting our claim that the produced set of parameters are both reasonably optimized and the resulting FF is generally stable. Also this suggests it is able to model Li–O systems in a broad range of temperatures and atom distances.
2. The LiO_2_ crystal is known to be unstable and decomposes at about room temperatures.^37^ Thus, the new GA/Opt-E seems to even predict this possibility, as seen from energy rise and discontinuity in *E*(*T*) graph, somewhat in contrast to the EEM prediction.
3. Similarly, lithium-ozonite crystal LiO_3_ structure is only obtained theoretically, suggesting its fragility and rare (if any) physical occurrence. The new GA/Opt-E FF does not predict its stability over the whole range as predicted by EEM, but this may as well be the expected behavior, based on mentioned experimental reasons.
4. As the final test of the obtained FF, here is taken to be the reaction of O_2_ diffusion at the lithium crystal slab. Remembering that we were starting from the ACKS2 FF hardly able to model Li crystal stability itself, the new FF should be considered well-behaved, at least at the level of comparison with the EEM FF taken as a sort of benchmark. There are certainly some differences in behavior of the two FFs during the simulation. Lacking the experimental data it is not easy to put preference to one or the other. The diffusivity constant of O_2_ in solid Li crystal may however be estimated based on the simulation time and knowledge of the crystal structure: lattice constant and the fact that it crystallizes in body-centered cubic (bcc) lattice. To the best of the authors’ knowledge no experimental or theoretical data for this value is available. The estimate here is based on the expression for the value for substitution or vacancy diffusion in the lattice as obtained from^38^
12 $$D=\frac{a^{2}\Gamma }{6}$$
  in units m^2^/s, where *a* is characteristic length of an individual diffusion molecule jump and Γ is the jump frequency. Thus, estimating jump distance to be of the order of two lithium atoms distance in its bcc lattice, i.e. of the value 3 Å if the lattice is of the order of 3.5 Å, and writing the total simulation time as *n* jumps per second, i.e.
13 t=n/Γ
  we obtain expression to estimate *D* from distance traveled by the oxygen (molecule) in the crystal.
  Number of jumps (or, better, the estimate of its minimum), *n* is estimated as so,
14 n=(totaldistance)/a
15 $$D=\frac{\left(\mathrm{total distance}\right)\times a}{6t}$$

From theoretical calculations available for niobium,^39^ which has a somewhat smaller unit cell but the same type of crystal lattice (bcc), we may attempt to estimate the distance we may expect the oxygen to travel in its diffusion through the lithium lattice. Taking *D* = 0.14 × 10^–6^ m^2^/s (O_2_ diffusivity in Nb_*x*_) and *t* = 12.5 ps, *a* = 3 Å, we get

(totaldistanceestimate)≈35 nm

which is about 100 unit cell distances. Although significantly larger, this still does not differ incomparably from the approximate distance of the furtherest oxygen measured in our simulations: 1.6 nm for EEM and 0.8 nm for GA/Opt-E. Also, the lower value obtained for GA/Opt-E does not necessarily signify a less reasonable result: the diffusivity coefficient is expected to be strongly dependent on the formation of vacancies in the metal lattice, which is estimated by lithium self-diffusivity. In the lithium crystal, the estimates in the literature vary from about 7 × 10^–9^ m^2^/s at the melting point to 6 × 10^–15^ m^2^/s at 293 K,^40,41^ to mention only two extreme examples. The reference data thus unfortunately vary in values over several orders of magnitude, but these values are significantly lower than the above prediction based on niobium. So, it may be concluded that both values obtained here for the FFs applied in final comparison, EEM as well as ACKS2-based GA/Opt-E, are reasonable. Lower limit for the constant of diffusion of oxygen in the lithium crystal at 300 K may thus be, by this overly simplified mechanistic approach, estimated from our MD results as

$$D_{\mathrm{EEM}}\approx 6.4\times 10^{-9}m^{2}/s$$

and

$$D_{\mathrm{GA}/\mathrm{Opt}-E}\approx 3.2\times 10^{-9}m^{2}/s$$

These should, of course, be seen as crude estimates only, as may be seen if all the complexity of the diffusion coefficient calculations from MD data is taken into account.^42^ To get a somewhat better fit, the more exact expression for obtaining the diffusion coefficient from MD data may be used.^43^ We write

16 $$D=\frac{1}{6}\lim_{t\to \infty }\langle \frac{\Delta {\left(r\left(t+\Delta t\right)-r\left(0\right)\right)}^{2}}{\Delta t}\rangle$$

and calculate the squared differences of positions divided by subsequent time intervals (100 intervals of 0.125 ps) from the obtained trajectory files. If such a method is applied, we get (following only the two oxygen atoms that penetrated into the crystal the most)

$$D_{EEM}\approx 1.3\times 10^{-6}m^{2}/s$$

and

$$D_{GA/Opt-E}\approx 1.7\times 10^{-6}m^{2}/s$$

(obtained as a mean value of the two most penetrating oxygen atoms in each simulation). Thus, both simulations predict a very similar oxygen diffusion coefficient in lithium, one comparable to the oxygen atom diffusion coefficient of *D* = 0.43·10^–6^ m^2^/s in a niobium bcc crystal reported in ref 44.

## 4 Conclusions

The previously developed EEM and ACKS2 *ReaxFF* FFs were validated through several methods, including comparison of charges obtained from the crystal and MD energy-minimized structures with *G16* DFT NPA charges. It is found that some discrepancy may be introduced by the parameters governing the MD energy structure minimization. Several further parameters are added to the reparametrization process, and the optimization is set using the *Optuna* Genetic Algorithm. The new optimized GA/Opt-E force field solves our initial criticism of the previous ACKS2 FF lithium crystal stability and also shows similar or superior performance compared to the well-established EEM FF, at least for the available experimental and theoretical data and for the investigated class of compounds. This has been supported by direct calculation of the oxygen diffusion coefficient and comparison to available data. In addition to the specific Li/O ACKS2 FF developed, the novel promising protocol, *Optuna* GA, enabling fast optimization of a large set of parameters, is introduced and used for the first time in *ReaxFF* parameter development.

## Supplementary Material









## Notes

SI1: all input structure files used for training and validation. SI1-A: Gaussian 16 inputs for structure and charge calculations for training. SI1-B: Gaussian 16 inputs for structure and charge calculations for validation. SI1-C: basis set for training GA/Opt and GA/Opt-E FFs. SI1-D: equation of state data: ReaxFF and Quantum Espresso inputs SI1-E: validation geometries and charges . SI4: trajectories for 12.5 ps MD simulations at 300 K using EEM and GA/Opt-E FFs (TXT)
SI2: complete description of detailed comparison of the available ACKS2 and EEM FFs, and the details of computational approach with the list of parameters used for optimization (PDF)
SI3: developed force field in.txt format (TXT)
SI4: Trajectories for 12.5 ps MD simulations at 300K using EEM and GA/Opt-E FFs (ZIP)
