# Supplementary material for: Charge Distribution and Lithium Oxide Stability Modeled by Reactive Force Field
Source: J Phys Chem A. 2025 Oct 13;129(43):10003–13. doi: 10.1021/acs.jpca.5c03998 (PMC12581134; doi:10.1021/acs.jpca.5c03998)
Supplement: Supplementary file 2 [file jp5c03998_si_002.pdf]

Supplementary information to the :

Charge Distribution and Lithium-oxide Stability Modeled by Reactive Force Field

Vjeran Gomzi*, Jakov Juvančić
University of Zagreb, Faculty of electrical engineering and computing,
Unska 3, 10 000 Zagreb, Croatia

vjeran.gomzi@unizg.fer.hr

Description of detailed comparison of the available ACKS2 and EEM FFs, using re-written *Python* code as well as the in-home compiled e-reactive *ReaxFF* fortran standalone code for several different study setups.

In addition to single unit cell considerations, 2x2x2 supercell has also been investigated, which enabled specific calculation setups described below.

When investigating 2x2x2 supercell, the list of performed calculations is as follows.

B. 2x2x2 supercell considerations:
1: *Python* code calculation of the supercell at their crystal structure using ACKS2 FF (py2A)
2: *Python* code calculation of the supercell at their crystal structure using EEM FF (py2E)
3: Full *ReaxFF* code calculation of the crystal at their crystal structure using ACKS2 FF (Rc2A)
4: Full *ReaxFF* code calculation of the crystal at their crystal structure using EEM FF (Rc2E)
5: Full *ReaxFF* code calculation of the optimized crystal structure using ACKS2 FF (Rco2A)
6: Full *ReaxFF* code calculation of the optimized crystal structure using EEM FF (Rco2E)
7: Full *ReaxFF* code calculation of the supercell at the crystal structure using ACKS2 FF (Rr2A)
8: Full *ReaxFF* code calculation of the supercell at the crystal structure using EEM FF (Rr2E)
9: Full *ReaxFF* code calculation of the supercell at the optimized crystal structure using ACKS2 FF (Rso2A)
10: Full *ReaxFF* code calculation of the supercell at the optimized crystal structure using EEM FF (Rso2E)

The distinction between calculation approaches 3-10 above should be given some more detailed explanation. The calculations 3 and 4 are done using the repeated supercell, but the atoms were held at their crystal positions. This is essentially the same as if the calculation is done on the initial crystal structure, apart from the fact that all of the charges (and positions) for the 2x2x2 structure are calculated. The calculations 5 and 6 are done using the same initial structure, but the structure was then energy minimized. So these refer to full crystal, but optimized. Calculations 7-10 are done exclusively on the supercell structure, atoms held in their initally obtained positions (7,8) or when structure optimization is done in *ReaxFF* (9,10).

The data presented in charts on Figure 2 is obtained by comparison with the same theoretical DFT method and using the essentially the same approach as done for single unit cell above.

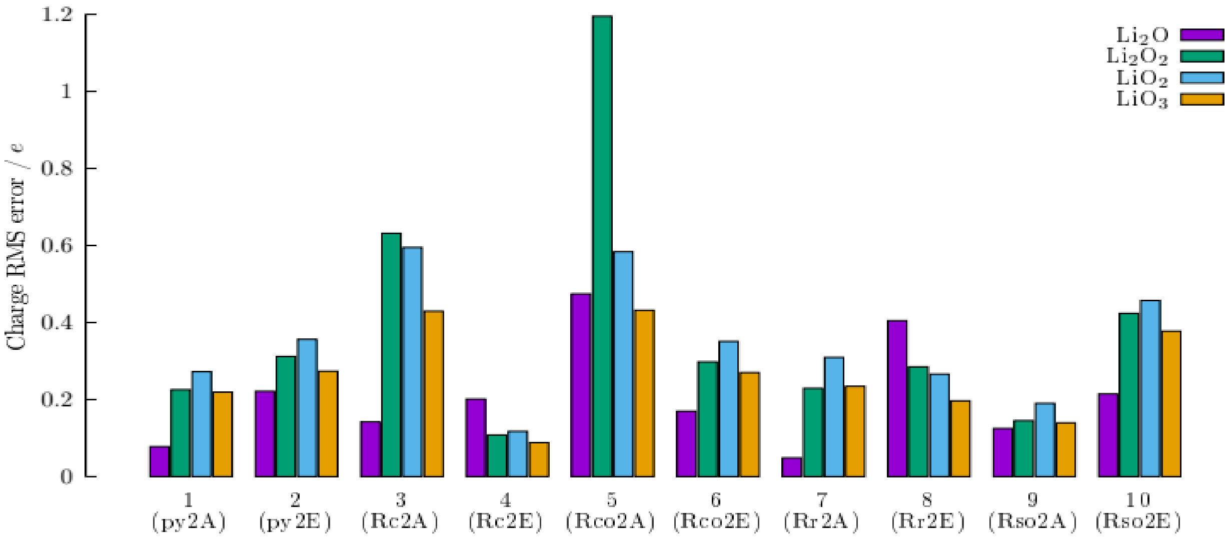

Figure S1: Comparison of charge distribution RMSEs from DFT theoretical values for different calculation protocols for all the lithium oxide structures in 2x2x2 supercells.

Analysis of Figure 2 leads to similar conclusions as drawn from unit cell investigation: inclusion of crystal environment still has relatively large impact, especially so for the ACKS2 calculation, and is larger if structures are allowed to optimize. This is again at the largest part the consequence of the fact that only 2x2x2 supercell structure (and not complete periodic crystal) is calculated by DFT and used for charge comparison. However, when supercell is optimized in *ReaxFF* MD using the ACKS2 charge model, it is seen that the charge distribution of most of the lithium oxide species are reproduced with high fidelity (chart 9 above). Comparison of charts 4 and 10 is interesting from the point of EEM approach validation: the charge distribution of EEM force-field yields much better results if applied on initial crystal structure, although comparison is done with the 2x2x2 DFT results. This may lead us to assume that other parameters governing energy minimization disturb structure which in turn leads to discrepancy seen in the chart 10 above. This is in line with the findings that structures are reasonably well reproduced using the ACKS2 FF developed in [S1]. However, even for the ACKS2, Li_2O crystal structure seems to be disturbed by optimization, as charges are reproduced better for supercell at their crystal positions for this oxide species (compare charts 7 and 9 above).

Detailed description of computational approach:

Required files:
ffield0 - initial *ffield* file to be re-optimized
control - standard control file for *ReaxFF* code (*ereac*)
geo - all the structures used in geometry training in .bgf format
exe - standard run file for standalone *ReaxFF* code
calculate_rmsd - a code snippet which calculates minimal difference of two .xyz structures
trainset.charges - DFT charges for a set of geometry training structures
trainset.energy - DFT energy differences for a set of structures in *geo* file (in kcal/mol)
opt.py - script governing parameter optimization in *Optuna* and modifying *ffield*
wrap.sh - shell script running *ereac*, *calculate_rmsd*, copying files and evaluating cost function

Optional files:
chgwt.wt - weights for individual structures in *trainset.charges*
enewt.wt - weights for each of relative energies in *trainset.energy*
geowt.wt - weights for optional scaling of structural differences

Required *Python* libraries:
subprocess, ***numpy***, ***Optuna***

The optimization is set up starting the *opt.py* which:

1. defines and runs Optuna study on a set of parameters from *ffield0*. This includes:

    1.1 defining initial parameters - *inipars*, *posit* and *minmax* - these are the three arrays which define the optimization: *inipars* are the parameters which we optimize as presented in the *ffield0* file, *posit* are the lines in *ffield0* in which these parameters are located, and *minmax* are the boundaries of search space (which we consistently chose to be ±40%)

    1.2 copying *ffield0* to *ffield* and modifying the parameters in *ffield* with current trial from the optimization process

    1.3 calling external script *wrap.sh* which returns the single numerical value which is dependent on parameters and which we require to be minimal.

2. The *ffield* file is now written using the current trial parameters and the output of *wrap.sh* is checked by *opt.py*. This output is obtained by the following steps:

    2.1 running *exe*, ie. *ereac*: this calculates all of the structures (output in *xmolout* file) and charges (output in *fort.56* file) using the current set of parameters in *ffield*. In *xmolout* file are also given the *ReaxFF* calculated energies.

    2.2 now the differences in structures are calculated using *calculate_rmsd* for all individual initial and final geometries, differences in charges from calculated and training charges and energies included in training are also compared. The script returns single floating number which is total weighted RMS sum of all the differences computed.

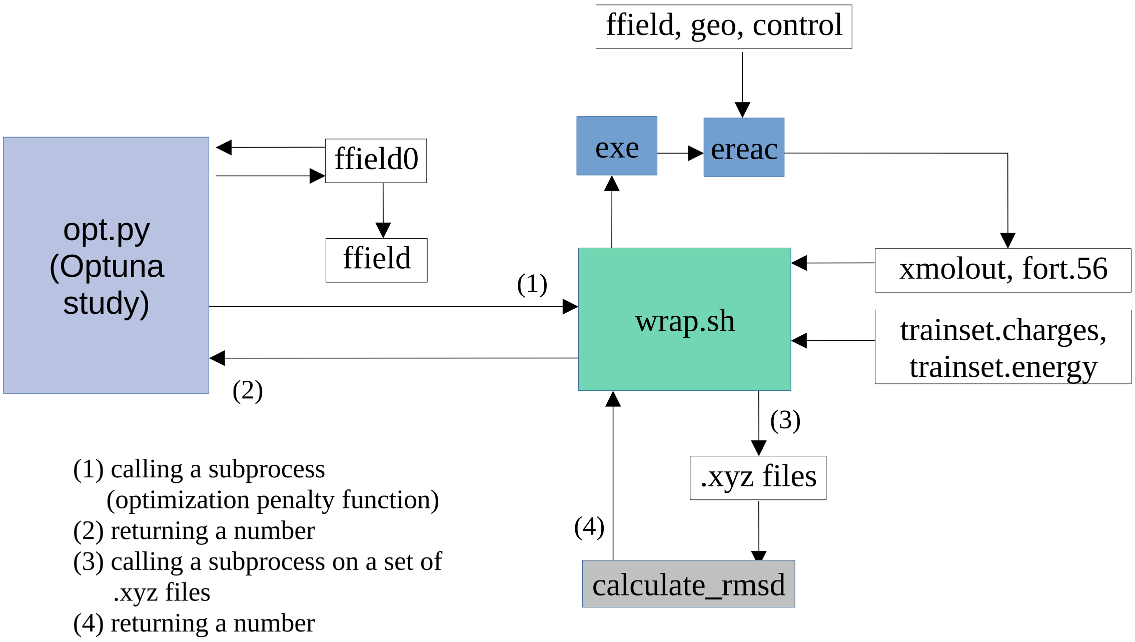

Figure S2: Diagram of applied code modules and their interlinkages. Colored elements are active programs, while empty boxes represent data files.

```
e-Reactive MD-force field: Li/Si/Al/O force field March 2020_JPCA
 39       ! Number of general parameters
   50.0000 !Overcoordination parameter
    9.5469 !Overcoordination parameter
   26.5405 !Valency angle conjugation parameter
    1.7224 !Triple bond stabilisation parameter
    6.8702 !Triple bond stabilisation parameter
   60.4850 !C2-correction
    1.0588 !Undercoordination parameter
    4.6000 !Triple bond stabilisation parameter
   12.1176 !Undercoordination parameter
   13.3056 !Undercoordination parameter
  -70.5044 !Triple bond stabilization energy
    0.0000 !Lower Taper-radius
   10.0000 !Upper Taper-radius
    2.8793 !Not used
   15.0000 !Valency undercoordination
    6.0891 !Valency angle/lone pair parameter
    1.0563 !Valency angle
    2.0384 !Valency angle parameter
    6.1431 !Not used
    6.9290 !Double bond/angle parameter
    0.3989 !Double bond/angle parameter: overcoord
    3.9954 !Double bond/angle parameter: overcoord
   -2.4837 !Not used
    5.7796 !Torsion/BO parameter
   10.0000 !Torsion overcoordination
    1.9487 !Torsion overcoordination
   -1.2327 !Conjugation 0 (not used)
    2.1645 !Conjugation
    1.5591 !vdWaals shielding
    0.0010 !Cutoff for bond order (*100)
    2.1365 !Valency angle conjugation parameter
    0.6991 !Overcoordination parameter
   50.0000 !Overcoordination parameter
    1.8512 !Valency/lone pair parameter
  234.3037 !eR-modif
   20.0000 !Not used
    5.0000 !Molecular energy (not used)
    0.0000 !Molecular energy (not used)
    2.6962 !Valency angle conjugation parameter
  6    ! Nr of atoms; cov.r; valency;a.m;Rvdw;Evdw;gammaEEM;cov.r2;#
               alfa;gammavdW;valency;Eunder;Eover;chiEEM;etaEEM;n.u.
               cov r3;Elp;Heat inc.;n.u.;n.u.;n.u.;n.u.
               ov/un;val1;n.u.;val3,vval4
 H    0.8930   1.0000   1.0080   1.3550   0.0930   0.8203  -0.1000   1.0000
      8.2230  33.2894   1.0000   0.0000 121.1250   3.7248   9.6093   1.0000
     -0.1000   0.0000  61.6606   3.0408   2.4197   0.0003   1.0698   0.0000
    -19.4571   4.2733   1.0338   1.0000   2.8793   1.0000   0.2000  12.0000
 O    1.2450   2.0000  15.9990   2.3890   0.0502   0.6318   1.0548   6.0000
      9.8993  13.8449   4.0000  37.5000 116.0768   9.0609   7.4275   2.0000
      0.9049   0.4056  59.0626   3.5027   0.7640   0.0021   3.4489   0.0000
     -4.0553   2.9000   1.0493   4.0000   2.9225   1.3000   0.2000  13.0000
 Si   2.2977   4.0000  28.0600   1.8329   0.1837   0.5947   1.2962   4.0000
     11.7318   5.2054   4.0000  21.7115 139.9309   4.2033   5.5558   0.0000
     -1.0000   0.0000 104.0000   9.0751  23.8188   0.8381   0.8563   0.0000
     -4.1684   2.0754   1.0338   4.0000   2.5791   1.4000   0.2000  13.0000
 Al   2.1967   3.0000  26.9820   2.3738   0.2328   0.4961  -1.6836   3.0000
      9.4002   1.6831   3.0000   0.0076  16.5151  -0.3343   6.5000   0.0000
     -1.0000   0.0000  78.4675  20.0000   0.2500   0.0000   0.8563   0.0000
    -23.1826   1.5000   1.0338   8.0000   2.5791   1.4000   0.2000  13.0000
```

```
 Li   1.9255   1.0000   6.9410   1.7722   0.1996   0.7511  -0.1000   1.0000
      9.9731   1.2063   1.0000   2.0000   3.0000  -6.0479   6.2978   0.0000
     -1.0000   2.0000  37.5000   5.4409   6.9107   0.1973   9.3096   0.0000
    -23.0012   2.2989   1.0338   1.0000   2.8103   1.3000   0.2000  13.0000
 X   -0.1000   2.0000   1.0080   2.0000   0.0000   1.0000  -0.1000   6.0000
     10.0000   2.5000   4.0000   0.0000   0.0000   5.0000 900.0000   0.0000
     -0.1000   0.0000 127.6226   8.7410  13.3640   0.6690   0.9745   0.0000
    -11.0000   2.7466   1.0338   6.2998   2.8793   0.0500   0.0010  10.0000
 15      ! Nr of bonds; Edis1;LPpen;n.u.;pbe1;pbo5;13corr;pbo6
                         pbe2;pbo3;pbo4;n.u.;pbo1;pbo2;ovcorr
  1  1 153.3934   0.0000   0.0000  -0.4600   0.0000   1.0000   6.0000   0.7300
         6.2500   1.0000   0.0000   1.0000  -0.0790   6.0552   0.0000   0.0000
  2  2 141.9324 145.0000  50.8293   1.2989  -0.1000   1.0000  29.7503   0.4910
         0.3451  -0.1055   9.0000   1.0000  -0.1225   5.5000   1.0000   0.0000
  1  2 160.0000   0.0000   0.0000  -0.5725   0.0000   1.0000   6.0000   0.5626
         1.1150   1.0000   0.0000   0.0000  -0.0920   4.2790   0.0000   0.0000
  1  3 101.1840   0.0000   0.0000  -0.1751   0.0000   1.0000   6.0000   1.1044
         7.3549   1.0000   0.0000   1.0000  -0.0450   7.9080   0.0000   0.0000
  2  3 273.4986   5.0000   0.0000  -0.3682  -0.3000   1.0000  36.0000   0.1000
        10.2378  -0.2158  28.6984   1.0000  -0.1451   8.0936   1.0000   0.0000
  3  3  70.3209  54.0531  30.0000   0.2206  -0.3000   1.0000  16.0000   0.0159
         0.0035  -0.8055   7.1248   1.0000  -0.0965   8.7733   0.0000   0.0000
  1  4  92.8579   0.0000   0.0000  -0.6528  -0.3000   0.0000  36.0000   0.1551
        10.0663  -0.3500  25.0000   1.0000  -0.0842   7.1758   0.0000   0.0000
  2  4 181.1998   0.0000   0.0000  -0.2276  -0.3000   0.0000  36.0000   0.1925
         0.2086  -0.3500  25.0000   1.0000  -0.2000   6.1462   0.0000   0.0000
  3  4   0.0000   0.0000   0.0000   1.0000   0.3000   0.0000  26.0000   1.0000
         0.5000   0.0000  12.0000   1.0000  -0.2000  10.0000   0.0000   0.0000
  4  4  34.0777   0.0000   0.0000   0.4832  -0.3000   0.0000  16.0000   0.5154
         6.4631  -0.4197  14.3085   1.0000  -0.1463   6.1608   0.0000   0.0000
  1  5  63.4649   0.0000   0.0000   0.0294   0.0000   0.0000   6.0000   0.4868
         0.3090   0.0000  12.0000   1.0000  -0.0800   5.1033   0.0000   0.0000
  2  5  76.8938  -0.0200   0.0000  -1.1429   0.3000   0.0000   6.0000   0.0631
         0.4301  -0.2500  11.9965   1.0000  -0.2188   6.6027   0.0000   0.0000
  3  5  23.2383   0.0000   0.0000   0.6079   0.3000   0.0000  26.0000   0.7696
         0.0781   0.0000  12.0000   1.0000  -0.1658   5.2531   0.0000   0.0000
  4  5   0.0000   0.0000   0.0000   1.0000   0.3000   0.0000  26.0000   1.0000
         0.5000   0.0000  12.0000   1.0000  -0.2000  10.0000   0.0000   0.0000
  5  5  45.0406   0.0000   0.0000   0.0064   0.3000   0.0000  26.0000   1.5411
         0.9010   0.0000  12.0000   1.0000  -0.1497   4.0597   0.0000   0.0000
 10     ! Nr of off-diagonal terms; Ediss;Ro;gamma;rsigma;rpi;rpi2
  1  2   0.0283   1.2885  10.9190   0.9215  -1.0000  -1.0000
  1  3   0.1035   1.3327  11.5963   1.3977  -1.0000  -1.0000
  2  3   0.1547   1.9047  10.7461   1.6501   1.3386  -1.0000
  1  4   0.0564   1.4937  12.0744   1.7276  -1.0000  -1.0000
  2  4   0.2017   1.8458  11.0700   1.6009  -1.0000  -1.0000
  3  4   0.1000   1.8500  10.3237  -1.0000  -1.0000  -1.0000
  1  5   0.2966   1.2550  10.2920   1.1989  -1.0000  -1.0000
  2  5   1.4736   1.4147   9.0283   1.5212  -1.0000   1.0000
  3  5   0.0800   2.0062  11.5806   1.7679   1.0000   1.0000
  4  5   0.0209   1.5000   9.0666  -1.0000   1.0000   1.0000
 36     ! Nr of angles;at1;at2;at3;Thetao,o;ka;kb;pv1;pv2;val(bo)
  1  1  1   0.0000  27.9213   5.8635   0.0000   0.0000   0.0000   1.0400
  2  2  2  80.7324  30.4554   0.9953   0.0000   1.6310  50.0000   1.0783
  1  2  2  75.6935  50.0000   2.0000   0.0000   1.0000   0.0000   1.1680
  1  2  1  85.8000   9.8453   2.2720   0.0000   2.8635   0.0000   1.5800
  2  1  2   0.0000  15.0000   2.8900   0.0000   0.0000   0.0000   2.8774
  1  1  2   0.0000   8.5744   3.0000   0.0000   0.0000   0.0000   1.0421
  3  3  3  78.5339  36.4328   1.0067   0.0000   0.1694   0.0000   1.6608
  1  3  3  68.5501  19.4239   2.3592   0.0000   0.2029   0.0000   1.0000
  1  3  1  70.7499  11.4850   4.6606   0.0000   1.5647   0.0000   1.0902
```

```
  2  3  3  91.0715  41.9975   3.5577   0.0000   1.6017   0.0000   3.0000
  1  3  2  73.6998  40.0000   1.8782   0.0000   4.0000   0.0000   1.1290
  2  3  2  80.6181  38.2534   7.5388   0.0000   0.0050   0.0000   2.4899
  2  3  3  91.0715  41.9975   3.5577   0.0000   1.6017   0.0000   3.0000
  3  2  3 103.8959  13.2049   2.7392   0.0000   1.6076   0.0000   1.9673
  1  2  3  90.0000   6.6857   1.6689   0.0000   2.5771   0.0000   1.0400
  2  2  3 107.9279  37.5298   8.0000   0.0000   1.7014   0.0000   2.2487
  1  1  3   0.0000   0.0100   1.0000   0.0000   1.0000   0.0000   2.0000
  3  1  3   0.0000   4.4216   0.8596   0.0000   0.9624   0.0000   1.0000
  2  1  3   0.0000   5.0000   1.0000   0.0000   1.0000   0.0000   1.2500
  2  1  4   0.0000   4.2750   1.0250   0.0000   1.3750   0.0000   1.4750
  1  1  4   0.0000   3.0000   1.0000   0.0000   1.0000   0.0000   1.2500
  4  1  4   0.0000  20.2391   0.1328   0.0000   2.9860   0.0000   1.0870
  1  2  4  88.1144  13.2143   1.5068   0.0000   3.0000   0.0000   1.0100
  2  2  4  34.4326  25.9544   5.1239   0.0000   2.7500   0.0000   1.7141
  4  2  4  20.7204  13.4875   4.0000   0.0000   0.6619   0.0000   1.4098
  1  4  1  67.4229   4.5148   5.9702   0.0000   3.0000   0.0000   2.6879
  1  4  2  41.8108  17.3800   2.6618   0.0000   0.7372   0.0000   1.0100
  2  4  2  69.3665  17.7549   3.1750   0.0000   3.0000   0.0000   1.1304
  1  4  4 180.0000 -26.7860   7.3549   0.0000   1.0000   0.0000   1.0252
  1  4  4  78.2279  37.6504   0.4809   0.0000   1.0000   0.0000   2.9475
  2  5  2  73.8444   7.3527   4.2355   0.0000   0.2652   0.0000   2.1889
  2  2  5  83.7686  29.7164   1.4275   0.0000   1.0000   0.0000   0.8509
  5  2  5  93.3484  19.0545   3.5779   0.0000   0.1000   0.0000   1.7236
  4  2  5  13.6525  12.0927   4.0000   0.0000   1.0000   0.0000   3.0818
  3  2  4  18.0953   5.3220   4.0000   0.0000   1.0000   0.0000   1.0139
  3  2  5  86.9269  18.2323   2.8071   0.0000   0.9102   0.0000   2.9549
  8    ! Nr of torsions;at1;at2;at3;at4;;V1;V2;V3;V2(BO);vconj;n.u;n
  1  2  2  2   0.8302  -4.0000  -0.7763  -2.5000  -1.0000   0.0000   0.0000
  2  2  2  2  -2.5000  -4.0000   1.0000  -2.5000  -1.0000   0.0000   0.0000
  0  1  1  0   0.0000   0.0000   0.0000   0.0000   0.0000   0.0000   0.0000
  0  1  2  0   0.0000   0.1000   0.0200  -2.5415   0.0000   0.0000   0.0000
  0  2  2  0   0.5511  25.4150   1.1330  -5.1903  -1.0000   0.0000   0.0000
  1  3  3  1   0.0000   0.0000   0.0640  -2.4426   0.0000   0.0000   0.0000
  1  3  3  3   0.0000   0.0000   0.1587  -2.4426   0.0000   0.0000   0.0000
  0  1  3  0   0.0000   0.0000   0.1200  -2.4847   0.0000   0.0000   0.0000
  1    ! Nr of hydrogen bonds;at1;at2;at3;Rhb;Dehb;vhb1
  2  1  2   2.1200   -3.5800   1.4500   19.5000
```

Figure S3: Marked positions of all the parameters included in optimization process in the initial file used for re-optimization, taken from [1].

References

[S1] K. A. O'Hearn, M. W. Swift, J. Liu; I. Magoulas, P. Piecuch, A. C. T. van Duin, H. M. Aktulga, Y. Qi, Optimization of the Reax force field for the lithium–oxygen system using a high fidelity charge model, *J. Chem. Phys.* 153 (2020) 084107. doi: doi.org/10.1063/5.0014406
